# What Influences Physicians’ Online Knowledge Sharing? A Stimulus–Response Perspective

**DOI:** 10.3389/fpsyg.2021.808432

**Published:** 2022-01-12

**Authors:** Xin Zhang, Xiaojia Dong, Xinxiang Xu, Jiahui Guo, Feng Guo

**Affiliations:** ^1^Management School, Tianjin Normal University, Tianjin, China; ^2^Business School, Nankai University, Tianjin, China; ^3^College of Management and Economics, Tianjin University, Tianjin, China

**Keywords:** online knowledge sharing, stimulus–response, online health platforms, online expertise, psychology

## Abstract

During the COVID-19 pandemic, online health platforms and physicians’ online knowledge sharing played an important role in public health crisis management and disease prevention. What influences physicians’ online knowledge sharing? From the psychological perspective of stimulus–response, this study aims to explore how patients’ visit and patients’ consultation influence physicians’ online knowledge sharing considering the contingent roles of physicians’ online expertise and online knowledge sharing experience. Based on 6-month panel data of 45,449 physician–month observations from an online health platform in China, the results indicate that both patients’ visit and patients’ consultation are positive related to physicians’ online knowledge sharing. Online expertise weakens the positive effect of patients’ consultation on physicians’ online knowledge sharing. Online knowledge sharing experience weakens the positive relationship between visit of patient and physicians’ online knowledge sharing, and enhances the positive relationship between patients’ consultation and physicians’ online knowledge sharing. This study contributes to the literatures about stimulus–response in psychology and knowledge sharing, and provides implications for practice.

## Introduction

The outbreak of COVID-19 has not only posed a severe threat to the healthy lives and wellbeing of people all over the world, but also caused significant challenges for health systems (Castelnuovo et al., 2020; Pan and Zhang, 2020; Luo et al., 2021). During the COVID-19 pandemic, online health platforms played an important role in public health crisis management and pandemic prediction (Zhao et al., 2020; Zhang et al., 2021). As a result of the rapid development of information technology (IT) and the huge demand for medical services, the delivery of health services on the internet has become increasingly popular (Hardey, 2001; Kvedar et al., 2014; Meng et al., 2021). Online healthcare can overcome geographic constraints and provide physicians with convenient access to information recipients (patients and their relatives); thus, an increasing number of physicians have been using online platforms to share their professional knowledge (Wu and Po, 2016; Zhang et al., 2019b). Physicians’ online knowledge sharing has also been found to alleviate unbalanced allocations of health resources (Kim and Mrotek, 2016), which is important for China given its large population and uneven distribution of health resources.

Physicians’ online knowledge sharing behavior has received extensive attention. Yan et al. (2016) applied the social exchange theory to investigate physicians’ online knowledge sharing, categorized the influential factors into benefit and cost, and proposed a benefit vs. cost knowledge sharing model. Zhang et al. (2017b) explored online knowledge sharing from the perspective of motivation theory and found that reputation, reciprocity, knowledge self-efficacy, and altruism were positively related to physicians’ online knowledge sharing intention. Meng et al. (2021) found that both online reputation and general knowledge sharing were positively related to specific knowledge sharing, and these relationships were moderated by patient involvement. However, few studies have explored physicians’ online knowledge sharing from the perspective of patients. The benefits of an online medical platform mainly derive from patients’ paid consultation, and patients’ participation is important to improve the operational proficiency of the platform; therefore, we cannot ignore the effect of patients’ participation on physicians’ behavior. It is important to explore physicians’ online knowledge sharing from the perspective of patients.

On an online medical platform, physicians’ behaviors usually depend on patients and they are stimulated process in psychology (Liu et al., 2016; Yan et al., 2016). According to the psychological framework of stimulus–response, patients’ visit and patients’ consultation are important indicators reflecting the reputation and popularity of physicians (Yang et al., 2015b; Liu et al., 2016), which is stimulus for physicians. In response to this stimulus, physicians may share knowledge online. However, the existing literature has not explored the effect of patients’ stimulus (patients’ visit, patients’ consultation) on physicians’ response (online knowledge sharing of physicians). To fill this research gap, this study expects that patients’ visit and patients’ consultation will induce physicians’ knowledge sharing. Accordingly, the first research question is presented as follows:

*Q1: How do patients’ visit and patients’ consultation influence physicians’ online knowledge sharing?*

User behavior regarding healthcare IT is not independent from its context (Zhang et al., 2021). To further investigate the boundaries of physicians’ online knowledge sharing, this study also explores whether the effects of patient visit and consultation are contingent on physicians’ online contexts. Physicians with high online expertise tend to have less freshness and interest in the platform; they will not pay attention to the stimuluses (patients’ visit and patients’ consultation) (Batson et al., 2002). Thus, online expertise may moderate the relationships between patients’ visit and patients’ consultation and physicians’ online knowledge sharing. In addition, previous studies have proposed that past behavioral experience can shape the human decision-making process (Chiu and Huang, 2015). Physicians with rich experience of sharing health knowledge online tend to form habits, which are unconscious processes that can influence the effects of conscious processes on decision outcomes (Honkanen et al., 2005; Chiu et al., 2012; Chiu and Huang, 2015). When knowledge sharing becomes a habit, physicians will regularly share knowledge on the platform rather than rely on patients’ visit and patients’ consultation for knowledge sharing. In this vein, online knowledge sharing experience may moderate the effects of patients’ visit and patients’ consultation on physicians’ online knowledge sharing. To explore the contingent factors that may affect the relationships between patients’ visit, patients’ consultation and physicians’ online knowledge sharing, our second research question is presented as follows:

*Q2: How are the relationships between patients’ visit, patients’ consultation and physicians’ online knowledge sharing moderated by physicians’ online expertise and online knowledge sharing experience?*

Drawing on the literature on the stimulus–response framework and knowledge sharing, a theoretical model associated with six hypotheses is developed. The hypotheses are tested using 6-month panel data with 45,449 physician–month observations from an online health platform in China. The results show that patients’ visit and patients’ consultation facilitate physicians’ online knowledge sharing. Online expertise and online knowledge sharing experience hinder the positive effect of patients’ visit on physicians’ online knowledge sharing, while online knowledge sharing experience intensifies the positive effect of patients’ consultation on physicians’ online knowledge sharing.

This study also contributes to the literature in several ways. First, it contributes to the psychological literature on stimulus–response by introducing the stimulus–response framework to track the mechanism of physicians’ online knowledge sharing. Based on the stimulus–response framework and literature of online knowledge sharing (Chen and Li, 2020; Meng et al., 2021), this paper uncovers the mechanism that patients’ visit and patients’ consultation benefit to physicians’ online knowledge sharing. Second, this study contributes to the literature on knowledge sharing by identifying and verifying the stimulated factors of physicians’ online knowledge sharing behavior from the perspective of patients. In response to calls that patients play a crucial role in value co-creation between physicians and patients (Van Oerle et al., 2016), our results reveal that both patients’ visit and patients’ consultation are important to physicians’ online knowledge sharing. Third, the study contributes to the literature on online knowledge sharing and expertise by revealing the contingency effects of online expertise and online knowledge sharing experience in the process of physicians’ online knowledge sharing. Behaviors of physicians and patients regarding healthcare IT is not independent from its context (Zhang et al., 2021), our empirical findings show that online expertise and online knowledge sharing experience indeed moderates the effects of patients’ visit and patients’ consultation on physicians’ online knowledge sharing.

The structure of our paper is organized as follows. The Section 2 presents the theory background and hypotheses. The Section 3 introduces the research methodology. The Section 4 illustrates the results. The discussion, theoretical contributions, practical contributions, limitations and future research, and conclusion are discussed in Section 5.

## Theory Background and Hypotheses

### Stimulus–Response Framework

The stimulus–response framework is a widely used psychological model (Reichl et al., 2006) that is introduced by Watson (1913). According to the framework, the complicated behaviors of humans are composed of stimulus and response (Watson, 1913; Giesen et al., 2020). Stimulus (S) refers to interior (individual) and exterior (environment) stimulation, while response (R) refers to the behavioral actions of humans in response to interrelated stimulus (Kim and Johnson, 2016; Luo et al., 2021).

The stimulus–response framework has been extensively applied in research into user online behaviors. Reichl et al. (2006) explored a charging mechanism for enhancing the quality of users’ experience by applying the stimulus–response framework. Li and Chang (2012) constructed an integrated model based on the stimulus–response framework to explore the factors influencing users’ continuous participation in virtual communities. The framework has also been used to investigate the relationship between online shopping festivals and consumer behavior (Xu et al., 2017; Liu et al., 2019). Chen and Li (2020) adopted the stimulus–response framework to explore the effect of product promotion strategies and atmosphere promotion strategies of consumers’ perception on their willingness to participate in online shopping.

The framework of stimulus–response offers a visualized framework that enables researchers to study the reasons for, and processes of, physicians’ online knowledge sharing behavior in online health communities (OHCs). Houston and Rothschild (1977) have classified stimulus into two categories. The first is stimulus from a specific object (e.g., patients’ visit), which becomes a consideration for the individual. In the context of OHCs, as a result of information asymmetry and intangibility (Arrow, 1963; Parasuraman et al., 1985), patients visit physicians’ homepages to obtain more information and assess whether the physicians meet their needs (Yang et al., 2015b). Physicians take the stimulus from patients’ visit into account, and this influences the physicians’ online knowledge sharing behavior. The second category is stimulus from the socio-psychology environment, which emphasizes the individual’s expectation regarding the presence or absence of another person (Houston and Rothschild, 1977). Feedback from others can act as stimulus for sharing knowledge (Oo Tha, 2014). OHCs between physicians and patients are accompanied by a series of dynamic interactions (Guo et al., 2017) (e.g., patients’ online consultation). Physicians provide health information during patients’ online consultations, and in return they receive feedback, professional recognition, respect, bonuses, and incentives (Yang and Ju, 2016; Liu Y. et al., 2020). Therefore, patients’ consultation stimulates physicians to respond to the expectation of patient presence, further influencing physicians’ online knowledge sharing behaviors.

Behaviors of physicians and patients regarding healthcare IT is not independent from its context (Zhang et al., 2021). The effects of patients’ visit and patients’ online consultation on physicians’ online knowledge sharing are dependent on context (e.g., online expertise and online knowledge sharing experience). Physicians with a high level of online expertise tend to realize strong online socialization through long-term sharing of knowledge as a result of their professional interests (Dodel and Mesch, 2018), which may affect the relationship between patients’ visit, patients’ online consultation and physicians’ online knowledge sharing. If physicians have rich experience of knowledge sharing (e.g., a large number of published articles), online knowledge sharing may be a habit and may become a habitual behavior. As an unconscious process, habits can affect the conscious process of making decisions (Chiu and Huang, 2015). However, few studies have investigated the moderating effects of online expertise and online knowledge sharing experience on the relationship between patients’ visit and patients’ online consultation (S) and physicians’ online knowledge sharing behavior (R). Online expertise and online knowledge sharing experience are crucial characteristics of physicians in OHCs; thus, it is important to explore their contingent effects.

In addition, physicians’ knowledge sharing behavior has been investigated from the perspective of charge or free of charge (Yang and Ju, 2016; Guo et al., 2017), but insufficient attention has been paid to patients’ involvement in stimulating physicians’ online knowledge sharing (Meng et al., 2021). The primary participants in an OHC platform (patients) play a crucial role in value co-creation between physicians and patients (Van Oerle et al., 2016). Attracting patients to participate in an OHC platform will better promote physicians’ online knowledge sharing behavior and increase the operational effectiveness of the OHC platform. The stimulus–response framework, as an important psychological model, reveals the effect of environmental factors on human’s behavior (Reichl et al., 2006; Li and Chang, 2012; Giesen et al., 2020). Applying the stimulus–response framework in online knowledge sharing facilitates to reveal the mechanism that physicians to learn about and interact with the patients is to receive a stimulus and respond to it accordingly and in real-time by sharing knowledge on the OHC platform. However, in the context of OHCs, few studies have investigated how stimulates from patients (patients’ visit, patients’ consultation) influence the response of physicians (online knowledge sharing). To address this gap, this study uses the stimulus–response framework to explore the effects of patients’ visit and patients’ online consultations (S) on physicians’ online knowledge sharing (R) and considers the contingent roles of physicians’ online expertise and online knowledge sharing experience.

### Patients’ Visit and Online Knowledge Sharing

Patients’ visit refers to the number of patients visit the physician’s homepage on the online health platform (Li et al., 2012). Online knowledge sharing refers to physicians providing free health and medical information on platforms that are available for viewers (Yan et al., 2016). Patients’ visit can have a positive effect on physicians’ online knowledge sharing for several reasons.

First, on the online health platform, the number of physicians’ homepage views indicates their service quality (Yang et al., 2015b). Physicians with more visits may have better service quality and will be welcomed by patients (Yang et al., 2015b). Physicians are motivated by what patients like, and they are motivated to interact with patients on the online health platform, which tends to induce their knowledge sharing on the platform (Zhang et al., 2019b). Second, patients’ visit has a positive effect on physicians’ online reputation (Cropanzano and Mitchell, 2005). The more visits, the better the physician’s online reputation, and online reputation plays a positive role in promoting knowledge sharing (Liu et al., 2016; Yan et al., 2016). Therefore, the more visits, the more knowledge sharing will be conducted by the physician. Finally, frequent patients’ visit to physicians’ homepages shows that patients are seeking medical knowledge and help from the articles shared by physicians. An increase in visits leads physicians to gradually understand the needs of patients. Therefore, to help their patients and serve their society (Luo et al., 2018), physicians share relevant medical knowledge on online health platforms. Based on the above argument, we propose the following hypothesis:

***H1:*** Patients’ visit *is positively related to physicians’ online knowledge sharing.*

### Patients’ Consultation and Online Knowledge Sharing

Patients’ consultation refers to a type of consultation in which physicians and patients are at different locations via an online health platform (Wu and Lu, 2017; Atanasova et al., 2018). In the context of online health platforms, the number of consultation is an important indicator that reflects physicians’ activity on the online health platform. Therefore, patients’ consultation can be an important factor affecting the online knowledge sharing of physicians.

On an online health platform, patients consult physicians when they encounter health problems (Guo et al., 2017). The more patients ask physicians about health problems, the easier it is for physicians to find common problems from the questions raised by patients (Ha and Longnecker, 2010). To save time, physicians can summarize the common questions they encounter and publish the answers on the online health platform. They can then use this knowledge to improve their professional knowledge capability, which increases the possibility of publishing relevant articles on the online health platform (Zhang et al., 2019a; Meng et al., 2021). In addition, a higher number of patient consultations show that physicians are interacting with patients on the platform more; that is, physicians are more involved in the platform. The more physicians participate, the more they share knowledge (Chang and Chuang, 2011; Liu and Jansen, 2017). Based on the above arguments, we propose the following hypothesis:

***H2:*** *Patients’ consultation is positively related to physicians’ online knowledge sharing.*

### The Moderating Effect of Online Expertise

Physicians’ online expertise refers to the online time and experience of physicians in using online platforms (Dodel and Mesch, 2018). Physicians’ high online expertise is accompanied by high levels of physicians’ assets, online time, and reputation (Kessler et al., 2015), which may moderate the effects of patients’ visit and patients’ consultation on physicians’ online knowledge sharing.

To a certain extent, online expertise reflects the physician’s assets, online time, and professional skills (Kessler et al., 2015; Dodel and Mesch, 2018). Physicians with stronger online expertise have longer online time and stronger professional skills. These physicians are accustomed to the operation of the platform, have less freshness and interest in the platform, and no longer pay attention to the number of visits and consultations (Batson et al., 2002). As a result of the reduced attention to the number of visits and consultations, the relationship between knowledge sharing and the number of visits and consultations is gradually weakened. In addition, physicians with a high level of online expertise have a high online reputation and old qualifications on the platform (Van Deursen et al., 2011). These physicians publish articles on the platform and share knowledge for incentive reasons instead of paying attention to the number of visits and consultations (Meng et al., 2021). For example, a senior physician of medicine said, “if knowledge sharing can save lives, it will be worth it in my life.” In this situation, the positive effect of visits and consultations of patients on physicians’ knowledge sharing will be weakened. Based on the above arguments, we propose the following hypotheses:

***H3:*** *Online expertise weakens the positive relationship between patients’ visit and physicians’ online knowledge sharing.*

***H4:*** *Online expertise weakens the positive relationship between patients’ consultation and physicians’ online knowledge sharing.*

### The Moderating Effect of Online Knowledge Sharing Experience

Online knowledge sharing experience refers to physicians’ past experience in contributing knowledge to the OHC (e.g., free and publicly available health articles shared by physicians (Yan et al., 2016). Online knowledge sharing experience reflects the situation of non-monetary benefits and physicians’ regular use of the platform (Zhang et al., 2017b), which may moderate the effect of patients’ visit and patients’ consultation on physicians’ online knowledge sharing.

OHCs aim to share and address health problems and provide support and encouragement to patients (Li et al., 2012; Guo et al., 2017). Physicians with extensive knowledge sharing experience participate in online knowledge sharing for non-monetary rather than monetary benefits (Zhang et al., 2017b). This is different from some online communities (e.g., online shopping platform), where people benefit by gaining monetary rewards (Papadopoulos et al., 2013; Park et al., 2014). Physicians with extensive knowledge sharing experience share knowledge for altruistic reasons. In this context, some extrinsic factors (e.g., patients’ visit and patients’ consultation) may not be the main drivers of knowledge sharing in OHCs (Chung, 2014), which weakens the positive relationships between patients’ visit, patients’ consultation, and physicians’ knowledge sharing.

Further, as a result of repetitive operation, previous knowledge sharing experiences may form a habit (Chiu et al., 2012). Habit, as an unconscious process, can influence the effects of conscious processes on decision outcomes (Chiu and Huang, 2015). So, knowledge sharing as an unconscious factor can shape a conscious decision-making process. Individuals with strong behavioral habits rely more on their past behavior rather than their cognitive evaluation, and vice versa (Honkanen et al., 2005; Chiu et al., 2012). According to Ouellette and Wood (1998), once a behavior becomes a habit, it is performed automatically and quickly, without attention. When knowledge sharing becomes a habit, physicians regularly share knowledge on the platform and do not rely on the patients’ visit and patients’ consultation for knowledge sharing. In this situation, online knowledge sharing experience will weaken the positive effect of patients’ visit and patients’ consultation on physicians’ online knowledge sharing. Based on the above arguments, we propose the following hypotheses:

***H5:*** *Online knowledge sharing experience weakens the positive relationship between patients’ visit and physicians’ online knowledge sharing.*

***H6:*** *Online knowledge sharing experience weakens the positive relationship between patients’ consultation and physicians’ online knowledge sharing.*

**FIGURE 1:**
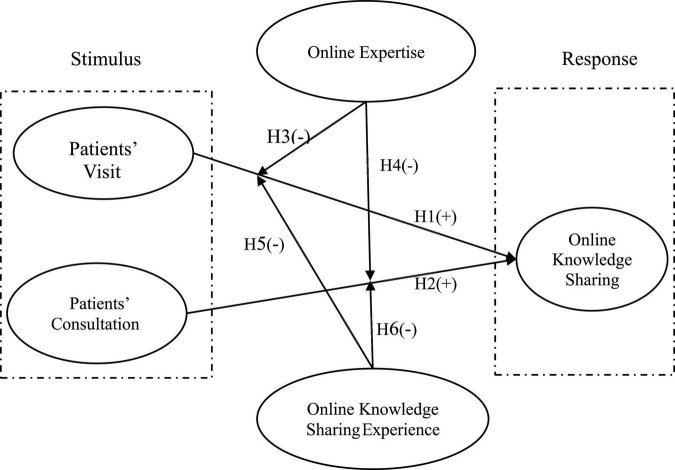
Research model.

In summary, the research model is presented in Figure 1.

## Methodology

### Data Connection

To avoid the self-reporting bias of surveys, this study applies objective data to test the hypotheses outlined above (Straub et al., 1995). The objective data were collected from *haodf.com* (“online good physicians” in English), a leading online health platform in China. This platform gathers more than 200,000 physicians from different hospitals throughout China and serves more than 58,000,000 patients online (Meng et al., 2021). The platform offers an ideal setting to explore physicians’ online knowledge sharing for the following reasons. First, it attracts many patients’ visit and consultations, which can induce physicians to share knowledge on the platform. Second, it enables physicians to share knowledge both publicly (without compensation) and privately (with compensation). Third, given the large number of participants, abundant data are generated about physicians’ websites and physician–patient interactions. We developed a Java-based web crawler to collect data from *haodf.com*. The article publications and website data statistics of 66,563 physicians over 6 months (February 2017 to July 2017) were collected. After removing some samples with incomplete data, we obtained 19,032 physicians for a total number of 45,449 physician–month observations.

### Measures

#### Dependent Variables

Online knowledge sharing (OKS) reflects physicians providing free health and medical information on platforms that are available for viewers (Yan et al., 2016). Based on previous studies (Kuang et al., 2019; Meng et al., 2021), online knowledge sharing was measured by the new number of shared free health articles in every month.

#### Independent Variables

Patients’ visit (PV) reflects the number of patients who visit the homepage of a physician on the health platform. We measured patients’ visit by the number of patients visiting a physician’s homepage. Patients’ consultation (PC) refers to a type of consultation in which physicians and patients are at different locations via online health platform (Wu and Lu, 2017; Atanasova et al., 2018). We measured patients’ consultation by the number of patients’ consultations on the health platform. Online expertise (OE) refers to online time and experience of physicians in using online platforms (Dodel and Mesch, 2018). We measured online expertise by the online time of the physician on the platform. Online knowledge sharing experience (OKSE) refers to physicians’ past experience in contributing knowledge to the OHCs. Following the suggestion of Meng et al. (2021), online knowledge sharing experience was measured by the number of free and publicly available health articles shared by physicians previously.

#### Control Variables

To ensure the model had a high level of precision, based on previous studies (Zhang et al., 2019b; Meng et al., 2021), this paper included control variables as follows. *Seniority* was measured by the professional title of the physician. *Gift* was measured by the number of online gifts from patients. *Thank-you* was measured by the number of online thank you letters from patients. *Vote* was measured by the number of votes received by the physician.

Given the magnitude of the variables, following the suggestion of Kafouros et al. (2015), we took the logarithm of all variables except seniority as our final measurement. Table 1 presents a summary of the variables.

**TABLE 1:** Variable description.

Variables		Description	Mean	*SD*	Min	Max
Dependent variables	Online knowledge sharing	New number of shared free health articles	0.226	0.645	0	7.169
Independent variables	Patients’ visit	Number of patients visiting a physician’s homepage	10.048	2.087	2.398	17.859
	Patients’ consultation	Number of patients’ consultations on the health platform	2.885	2.499	0	10.667
	Online expertise	Opening time of physician the platform	6.789	1.067	1.792	8.030
	Online knowledge sharing experience	Number free and publicly available health articles shared by physicians previous	0.890	1.250	0	7.551
Control variables	Seniority	Professional title of the physician	2.804	0.970	1	4
	Gift	Number of Online gifts from patients	1.016	1.429	0	7.920
	Thank-you	Number of online thank-you letters from patients	0.759	1.037	0	6.066
	Vote	Number of votes received by the physician	1.612	1.312	0	6.911

### Data Analysis

To better understand the relationship between patients’ visit (PV), patients’ consultation (PC), online expertise (OE), online knowledge sharing experience (OKSE), and online knowledge sharing (OKS), we applied a moderated-model analysis. In line with previous studies (Wan and Sanders, 2017; Zhang et al., 2019b), a three-model system to analyze the relationships among the variables was presented as follows:


$$\begin{array}{lll} {\text{OKS}}_{it} & = & \alpha_{0}+\alpha_{1}Seniority_{it}+\alpha_{2}Gift_{it}+\alpha_{3}Thank-you_{it}\\ & & +\alpha_{4}Vote_{it}+\alpha_{5}PV_{it}+\alpha_{6}{\text{PC}}_{it}+\mu_{it}\\ {\text{OKS}}_{it} & = & \beta_{0}+\beta_{1}Seniority_{it}+\beta_{2}Gift_{it}+\beta_{3}Thank-you_{it}\\ & & +\beta_{4}Vote_{it}+\beta_{5}PV_{it}+\beta_{6}{\text{PC}}_{it}+\beta_{7}{\text{OE}}_{it}+\beta_{8}PV_{it}\\ & & \times {\text{OE}}_{it}+\beta_{9}PC_{it}\times {\text{OE}}_{it}+\varepsilon_{it}\\ {\text{OKS}}_{it} & = & \gamma_{0}+\gamma_{1}Seniority_{it}+\gamma_{2}Gift_{it}+\gamma_{3}Thank-you_{it}\\ & & +\gamma_{4}Vote_{it}+\gamma_{5}PV_{it}+\gamma_{6}{\text{PC}}_{it}+\gamma_{7}{\text{OKSE}}_{it}\\ & & +\gamma_{8}PV_{it}\times {\text{OKSE}}_{it}+\gamma_{9}PC_{it}\times {\text{OKSE}}_{it}+\varphi_{it} \end{array}$$

Where i = 1, 2, 3, ⋯, N indicate the numbers of observations; α_0_ to α_6_, β_0_ to β_10_, γ_0_ to γ_11_ are the parameters to be estimated in the three equations; and μ_*it*_,ε_*it*_,φ_*it*_ are the error terms in the three equations.

Previous studies have noted that the ordinary least squares regression model is inefficient and is accompanied by estimated bias if the testing excludes time effects (Lee et al., 2014). Following the method for panel data applied by Samila and Sorenson (2010) and Lee et al. (2014), this paper applied the fixed-effects model to investigate the relationship between the explaining variables and the explained variables.

## Results

Table 2 presents the correlation matrix for the study measures. Since our study involved moderating effects, following existing recommendations and recent empirical studies (Cohen et al., 2003; Fischer et al., 2019; Meng et al., 2021), we applied hierarchical regression to test the hypotheses. In line with the conclusions of Brambor et al. (2006) and Hayes and Matthes (2009), centering would not offer any new or more accurate information, and would help us to overcome any problem with multicollinearity; thus, we did not mean center predictor variables. The tests of the hypotheses are presented in Table 3.

**TABLE 2:** Correlation matrix.

Variables	1	2	3	4	5	6	7	8	9
1.Online knowledge sharing	1.000								
2.Patients’ visit	0.260	1.000							
3.Patients’ consultation	0.301	0.823	1.000						
4.Online expertise	0.505	0.652	0.642	1.000					
5.Online knowledge sharing experience	0.070	0.712	0.282	0.286	1.000				
6.Seniority	0.038	0.350	0.175	0.160	0.357	1.000			
7.Gift	0.279	0.693	0.814	0.569	0.229	0.178	1.000		
8.Thank-you	0.217	0.627	0.682	0.462	0.260	0.288	0.768	1.000	
9.Vote	0.202	0.689	0.689	0.446	0.363	0.411	0.727	0.878	1.000

**TABLE 3:** Results of hierarchical regression.

DV: Online knowledge sharing	Model 1	Model 2	Model 3
	Coefficient (Standard error)	Coefficient (Standard error)	Coefficient (Standard error)
Patients’ visit (PV)	0.015*** (0.003)	0.118*** (0.018)	−0.014*** (0.003)
Patients’ consultation (PC)	0.056*** (0.003)	0.028* (0.014)	−0.010*** (0.003)
Online expertise (OE)		−0.033 (0.018)	
Online knowledge sharing experience (OKSE)			0.801*** (0.016)
VP × OE		−0.006* (0.002)	
CP × OE		−0.0005 (0.002)	
VP × OKSE			−0.062*** (0.002)
CP × OKSE			0.046*** (0.002)
Seniority	−0.008* (0.003)	−0.007 (0.003)	−0.004 (0.003)
Gift	0.046*** (0.004)	0.041*** (0.004)	−0.001 (0.004)
Thank-you	0.021*** (0.006)	0.020** (0.006)	0.008 (0.006)
Vote	−0.040*** (0.005)	−0.040*** (0.005)	−0.002 (0.005)
Constant	−0.057** (0.021)	−0.386** (0.122)	0.136*** (0.023)
R square	0.098	0.102	0.284

*p < 0.05, **p < 0.01, ***p < 0.001 (2-tailed test).

In Model 1, we regressed online knowledge sharing on patients’ visit and patients’ consultation. The results indicated that patients’ visit (*b* = 0.015, *p* < 0.001) and patients’ consultation (*b* = 0.056, *p* < 0.001) were positive and significantly related to online knowledge sharing. Thus, H1 and H2 are supported. For the control variables, the effects of seniority (*b* = −0.008, *p* < 0.050) and vote (*b* = −0.039, *p* < 0.001) were negative and significant, while the effects of gift (*b* = 0.047, *p* < 0.001) and thank-you (*b* = 0.020, *p* < 0.010) were positive and significant.

**FIGURE 2:**
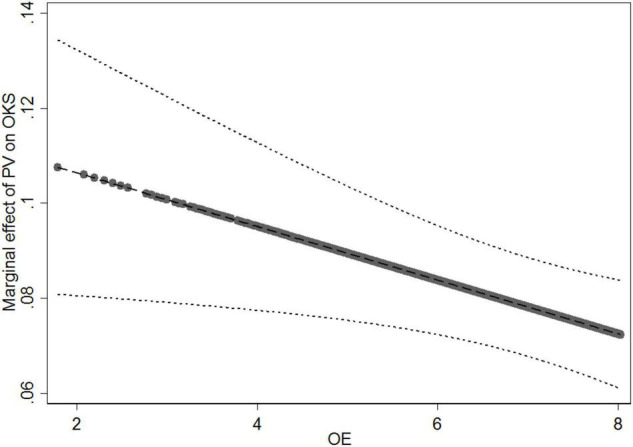
Moderating effect of online expertise (OE) on the relationship between patients’ visit (PV) and online knowledge sharing (OKS).

In Model 2, to test the moderating effects of online expertise, we computed the interaction terms between patients’ visit (PV), patients’ consultation (PC), and online expertise (OE), and then entered them into the regression equation after the control variables and the direct effects. The results showed that the coefficient of the interaction term (PV × OE) was negative and significant (*b* = −0.006, *p* < 0.050). Following the suggestion of Meyer et al. (2017), we calculated and plotted the marginal effect of patients’ visit on online knowledge sharing at different levels of online expertise (Figure 2). The results indicated that as the values of online expertise increased from 1.792 to 8.030, the slope of the relationship between patients’ visit and online knowledge sharing becomes flatter. It suggest that online expertise weakens the positive effect of patients’ visit on online knowledge sharing. Thus, H3 is supported. The relationship between the interaction term (PC × OE) and online knowledge sharing was not statistically significant (*b* = −0.0005, *p* > 0.050). Thus, H4 is not supported.

**FIGURE 3:**
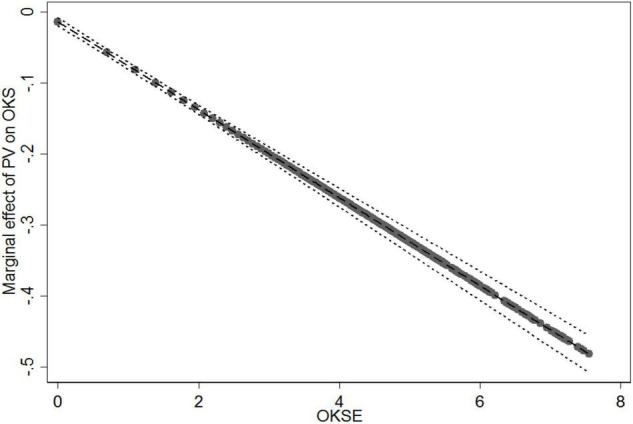
Moderating effect of online knowledge sharing experience (OKSE) on the relationship between patients’ visit (PV) and online knowledge sharing (OKS).

**FIGURE 4:**
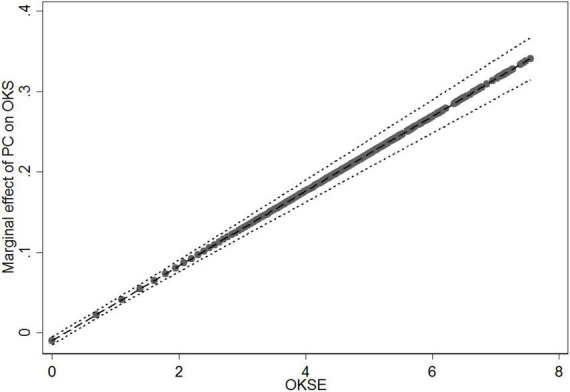
Moderating effect of online knowledge sharing experience (OKSE) on the relationship between patients’ consultation (PC) and online knowledge sharing (OKS).

In Model 3, to test the moderating effects of online knowledge sharing experience, we computed the interaction terms between patients’ visit (PV), patients’ consultation (PC), and online knowledge sharing experience (OKSE), and then entered them into the regression equation after the control variables and the direct effects. In Model 3, the relationship between the interaction term (PV × OKSE) and online knowledge sharing were statistically significant (*b* = −0.062, *p* < 0.001). We plotted the marginal effect of patients’ visit on online knowledge sharing at different levels of online knowledge sharing experience (Figure 3). The results show that as the values of online knowledge sharing experience increase from 0 to 7.551, the slope of the relationship between patients’ visit and online knowledge sharing becomes flatter. In other words, online knowledge sharing experience reduces the positive effect of patients’ visit on online knowledge sharing. Thus, H5 is supported. The coefficients of the interaction term (PC × OKSE) were statistically positive and significant (*b* = 0.046, *p* < 0.001). We plotted the marginal effect of patients’ consultation on online knowledge sharing at different levels of online knowledge sharing experience (Figure 4). Figure 4 demonstrates that as the values of online knowledge sharing experience increased from 0 to 7.551, the slope of the relationship between patients’ consultation and online knowledge sharing becomes steeper. It means that online knowledge sharing experience enhances the positive effect of patients’ consultation on online knowledge sharing. These results are contrary to our hypothesis. Thus, H6 is not supported.

To check the robustness of our results, following the suggestion of previous studies (Wiener and Lee, 2020; Chin et al., 2021), we conducted supplementary analysis with random effects models to test our hypotheses. The results are presented in Table 4. Model 4 indicated that patients’ visit (*b* = 0.015, *p* < 0.001) and patients’ consultation (*b* = 0.056, *p* < 0.001) were positively and significantly related to online knowledge sharing. Thus, H1 and H2 are supported. Model 5 showed that the coefficient of the interaction term (PV × OE) was negative and significant (*b* = −0.007, *p* < 0.010), while the coefficient of the interaction term (PC × OE) was insignificant (*b* = −0.0004, *p* > 0.050). Therefore, H3 is supported but H4 is not supported. Model 6 indicated that the interaction term (PV × OKSE) was negatively and significantly related to online knowledge sharing (*b* = −0.062, *p* < 0.010), while the interaction term (PC × OKSE) was positively and significantly related to online knowledge sharing (*b* = 0.046, *p* < 0.010). Thus, H5 is supported but H6 is not supported. In summary, the results of random effects are consistent with those of fixed effects, and our results are robust.

**TABLE 4:** Results of robust test.

DV: Online knowledge sharing	Model 1	Model 2	Model 3
	Coefficient (Standard error)	Coefficient (Standard error)	Coefficient (Standard error)
Patients’ visit (PV)	0.015*** (0.003)	0.126*** (0.018)	−0.013*** (0.003)
Patients’ consultation (PC)	0.056*** (0.003)	0.023 (0.014)	−0.010*** (0.003)
Online expertise (OE)		−0.018 (0.018)	
Online knowledge sharing experience (OKSE)			0.806*** (0.016)
VP × OE		−0.007* (0.002)	
CP × OE		−0.0004 (0.002)	
VP × OKSE			−0.062*** (0.002)
CP × OKSE			0.046*** (0.002)
Seniority	−0.008* (0.003)	−0.006 (0.003)	−0.004 (0.003)
Gift	0.047*** (0.004)	0.043*** (0.004)	−0.0001 (0.004)
Thank-you	0.020** (0.006)	0.020** (0.006)	0.007 (0.006)
Vote	−0.039*** (0.005)	−0.039*** (0.005)	−0.0004 (0.005)
Constant	−0.066** (0.021)	−0.471** (0.122)	0.125*** (0.023)
R square	0.098	0.102	0.283

*p < 0.05, **p < 0.01, ***p < 0.001 (2-tailed test).

## Discussion and Conclusion

### Discussion

During the COVID-19 pandemic, online health platforms and physicians’ online knowledge sharing has played an important role in public health crisis management and disease prevention (Zhang et al., 2021). This study, based on the stimulus–response framework in psychology, investigated the reasons for, and processes of, physicians’ online knowledge sharing and resulted in four significant key findings.

First, this study found support for the stimulus–response hypothesis. Patients’ visit and online consultations are positively related to physicians’ online knowledge sharing. This means that patients are able to stimulate physicians’ online knowledge sharing (e.g., publishing online health articles). The number of patients’ visit is an important indicator evaluating the online reputation of physicians (Meng et al., 2021), which is a vital factor affecting their knowledge sharing behavior (Yan et al., 2016; Zhang et al., 2017b; Park and Gabbard, 2018). Patients’ consultation reflects interactions between physicians and patients, and physicians actively participate in the process; the more physicians participate, the more they share knowledge (Chang and Chuang, 2011; Liu and Jansen, 2017).

Second, this study verified the moderating effect of online expertise. Physicians’ online expertise reflects their online skills and experience in using online platforms (Dodel and Mesch, 2018). Physicians with a high level of online expertise feel less freshness and interest in the platform (Batson et al., 2002); in this context, they no longer pay attention to the patients’ visit in online knowledge sharing. In this vein, online expertise weakens the positive relationship between patients’ visit and online knowledge sharing. However, the hypothesis that online expertise alleviates the effect of patients’ consultation on physicians’ online knowledge sharing is not supported. One possible explanation is that patients’ consultation involves frequent interactions between physicians and patients, and physicians need to focus on it (Yang et al., 2015a; Liu S. et al., 2020). Regardless of the level of online expertise, all physicians tend to attach importance to patient consultations; thus, the relationship between patient consultations and physicians’ online knowledge sharing is almost not affected by online expertise.

Finally, the moderating effect of online knowledge sharing experience was also confirmed. This study finds that online knowledge sharing experience weakens the positive relationship between patients’ visit and physicians’ online knowledge sharing, and enhances the positive relationship between patients’ consultation and physicians’ online knowledge sharing. Physicians with online knowledge sharing experience tend to form the habit of online knowledge sharing behavior and ignore the stimulus effect of patients’ visit. It is indicates an individual unconscious process reducing the influence of the conscious process, consistent with prior literature (Chiu and Huang, 2015; Zhang et al., 2017a). However, online knowledge sharing expertise heightens the stimulus effect of patients’ online consultation. One possible explanation is that physicians with online knowledge sharing experience discover common knowledge via patients’ consultation (Li et al., 2019). In this context, patients’ consultation enables physicians to summarize the common knowledge and share it online, thereby enhancing the positive relationship between patients’ consultation and physicians’ online knowledge sharing.

### Theoretical Contributions

This study makes several theoretical contributions to the literature. First, this study extends the stimulus–response literature of psychology by introducing the stimulus–response framework to track the mechanism of physicians’ online knowledge sharing. The stimulus–response framework offers a visualized framework to help researchers study the reasons for, and processes of, physicians’ online knowledge sharing behavior in OHCs. In the context of OHCs, patients’ visit and online consultations both have stimulating effects on physicians’ online behavior response. Although much of the research based on the stimulus–response framework has been conducted to study online user behavior (Reichl et al., 2006; Xu et al., 2017; Chen and Li, 2020), to our knowledge, the framework has not been applied to investigate physicians’ online knowledge sharing behavior in the context of OHCs. Thus, this study extends the stimulus–response framework literature by introducing the framework to investigate physicians’ online knowledge sharing mechanism.

Second, this study extends online knowledge sharing literature by revealing the stimulus mechanism of patients’ behaviors on physicians’ behaviors. The primary participants in an OHC platform (patients) play a crucial role in value co-creation between physicians and patients (Van Oerle et al., 2016). Attracting patients to participate in the OHC platform will better promote physicians’ online knowledge sharing behavior and increase the operational effectiveness of the OHC platform. Although physicians’ online knowledge sharing behavior has been widely explored, existing literature mainly focuses on the perspective of charge or free of charge (Yang and Ju, 2016; Guo et al., 2017). Few studies have explored how patients’ behavior stimulates physicians’ behavior from the perspective of patients (Meng et al., 2021). Our results reveal that both patients’ visit and patients’ consultation are positively related to physicians’ online knowledge sharing, which provides a new perspective for exploring how patients’ behaviors influence physicians’ behaviors on online medical platforms.

Third, this study enriches the online expertise and online knowledge sharing literature by uncovering the contingent effect of online expertise in the process of physicians’ online knowledge sharing. The expertise of physicians is an important contingent factor in exploring their online behavior, but little attention has been paid to their online expertise (Guo et al., 2017; Luo et al., 2018). Our study finds that online expertise negatively moderates the effect of patients’ visit on physicians’ online knowledge sharing. Physicians with high online expertise tend to neglect stimulus from patients’ visit because of their interests (Dodel and Mesch, 2018); thus, the positive relationship between patients’ visit and physicians’ online knowledge sharing is weakened. Therefore, our discoveries enrich the studies of online expertise and online knowledge sharing.

Finally, this study enriches online knowledge sharing literature by uncovering the contingent effect of online knowledge sharing experience in the process of physicians’ online knowledge sharing. Physicians with high levels of online knowledge sharing experience are likely to form habits. This reduced the stimulus effect of patients’ visit, which indicates an individual unconscious process reducing the influence of the conscious process, consistent with prior studies (Chiu and Huang, 2015; Zhang et al., 2017a). Online knowledge sharing experience helps physicians discover common knowledge via patients’ consultation (Li et al., 2019). In this context, patients’ consultation facilitates physicians to summarize the common knowledge and share it online. Thus, we find that online knowledge sharing experience strengthens the positive relationship between patients’ consultation and physicians’ online knowledge sharing. Hence, our study enriches the literature of online knowledge sharing by uncovering the different moderating effects of online knowledge sharing experience.

### Practical Contributions

This study has several practical implications for OHC practitioners and platform managers. First, our results show that physicians’ online knowledge sharing is positively promoted by patients’ visit and online consultations. Patients can benefit from physicians’ online knowledge sharing—for example, by obtaining free health articles, increasing their visits to OHCs (Meng et al., 2021), and then gaining social (Johnston et al., 2013) and emotional support (Yan and Tan, 2014). As a result, patients should stimulate physicians to share knowledge with the aid of more visits to physicians’ homepages and more online consultations, thereby achieving value co-creation.

Second, physicians should understand their decision-making processes in terms of knowledge sharing. This study finds that physicians’ online expertise and knowledge sharing experience play significant moderating roles in their online knowledge sharing. Hence, as important participants in OHCs, different groups of physicians should be aware of how their levels of online experience affect their sharing decision-making to make better decisions. For example, physicians with a low level of online expertise feel more freshness and interest in the platform, they often pay more attention to the patients’ visit in online knowledge sharing. These physicians should exert their subjective initiative and actively publish free articles for attracting more patients to visit their homepages, which stimulating more online knowledge sharing in turn and creating a virtuous circle.

Finally, platform managers can use diverse strategies to stimulate different physician groups. Our results show that the stimulus effect of patients’ visit is weakened by physicians’ online knowledge sharing experience and online expertise. Thus, managers can introduce measures to guide more patients to browse the homepages of physicians with low knowledge sharing experience and online expertise, thereby stimulating them to share knowledge online. For example, the platform can push physicians with low level of online expertise or few published articles to patients, by launching preferential activities such as browsing and punching in, to increase the visits of physicians’ homepages. Meanwhile, the stimulus effect of patients’ consultation is strengthened by physicians’ online knowledge sharing experience. Therefore, managers can take measures to guide patients to consult physicians with rich experience of online knowledge sharing for stimulating physicians to share knowledge. For example, the platform can recommend physicians who have published many articles to patients in need of consultation. Moreover, managers should emphasize the potential benefits (e.g., social and economic returns) of online knowledge sharing to encourage physicians to contribute persistently to OHCs (e.g., by publishing online health articles).

### Limitations and Future Research

Although this study has produced interesting findings and contributed to both theory and practice, it has several limitations. First, the results of the study are based on data in the Chinese context, which may limit the generalization to other countries (Wu et al., 2019; Zhao et al., 2020; Meng et al., 2021). Future research should use data from diverse countries to verify the validity of our results. Second, this study only used online expertise and online knowledge sharing experience as moderators; however, other factors could be used in the study of physicians’ online knowledge sharing, such as physicians’ offline seniority and information uncertainty (Zhang et al., 2019b; Liu Y. et al., 2020). Finally, the research model in this study does not contain mediators. In fact, physicians’ affective states (e.g., empathy and pleasure) caused by stimulus (Kim and Johnson, 2016; Luo et al., 2021) may affect their response in online knowledge sharing. Future research could introduce physicians’ affective states as mediators to investigate physicians’ online knowledge sharing.

## Conclusion

The COVID-19 not only causes significant challenges for health systems and economic recession, but also influence physicians’ online knowledge sharing. This study investigates physicians’ online knowledge sharing by applying the framework of stimulus–response in psychology and considers the contingency effect of physicians’ online expertise and online knowledge sharing experience. Based on the 6-month panel data of 45,449 physician–month observations from an online health platform in China, this study uncovers that patients’ visit and patients’ consultation benefit physicians’ online knowledge sharing. Meanwhile, online expertise and online knowledge sharing experience impede the positive relationship between patients’ visit and physicians’ online knowledge sharing, while online knowledge sharing experience enhances the positive relationship between patients’ consultation and physicians’ online knowledge sharing. Our study also has theoretical contributions to the literature of stimulus–response and online knowledge sharing, and practical implications to OHC practitioners and platform managers.

## Data Availability Statement

The raw data supporting the conclusions of this article will be made available by the authors, without undue reservation.

## Author Contributions

All authors listed have made a substantial, direct, and intellectual contribution to the work, and approved it for publication.

## Conflict of Interest

The authors declare that the research was conducted in the absence of any commercial or financial relationships that could be construed as a potential conflict of interest.

## Publisher’s Note

All claims expressed in this article are solely those of the authors and do not necessarily represent those of their affiliated organizations, or those of the publisher, the editors and the reviewers. Any product that may be evaluated in this article, or claim that may be made by its manufacturer, is not guaranteed or endorsed by the publisher.

## References

[B1] Arrow K. J. (1963). Uncertainty and the welfare economics of medical care. *Am. Econ. Rev.* 53 941–973.

[B2] Atanasova S. Kamin T. Petrič G. (2018). The benefits and challenges of online professional-patient interaction: comparing views between users and health professional moderators in an online health community. *Comput. Hum. Behav.* 83 106–118. 10.1016/j.chb.2018.01.031

[B3] Batson C. D. Ahmad N. Tsang J. A. (2002). Four motives for community involvement. *J. Soc. Issues.* 58 429–445.

[B4] Brambor T. Clark W. R. Golder M. (2006). Understanding interaction models: improving empirical analyses. *Polit. Anal.* 14 63–82. 10.1093/pan/mpi014

[B5] Castelnuovo G. De Giorgio A. Manzoni G. M. Treadway D. C. Mohiyeddini C. (2020). Psychological, behavioral, and interpersonal effects and clinical implications for health systems of the coronavirus (COVID-19) pandemic: a call for research. *Front. Psychol.* 11:2146. 10.3389/fpsyg.2020.02146 33071842 PMC7541698

[B6] Chang H. H. Chuang S.-S. (2011). Social capital and individual motivations on knowledge sharing: participant involvement as a moderator. *Inf. Manag.* 48 9–18. 10.1016/j.im.2010.11.001

[B7] Chen C. Li X. (2020). The effect of online shopping festival promotion strategies on consumer participation intention. *Ind. Manag. Data Syst.* 120 2375–2395. 10.1108/imds-11-2019-0628

[B8] Chin T. Wang W. Yang M. Duan Y. Chen Y. (2021). The moderating effect of managerial discretion on blockchain technology and the firms’ innovation quality: evidence from Chinese manufacturing firms. *Int. J. Prod. Econ.* 240:108219. 10.1016/j.ijpe.2021.108219

[B9] Chiu C.-M. Huang H.-Y. (2015). Examining the antecedents of user gratification and its effects on individuals’ social network services usage: the moderating role of habit. *Eur. J. Inform. Syst.* 24 411–430. 10.1057/ejis.2014.9

[B10] Chiu C.-M. Hsu M.-H. Lai H. Chang C.-M. (2012). Re-examining the influence of trust on online repeat purchase intention: the moderating role of habit and its antecedents. *Decis. Support Syst.* 53 835–845. 10.1016/j.dss.2012.05.021

[B11] Chung J. E. (2014). Social networking in online support groups for health: how online social networking benefits patients. *J. Health Commun.* 19 639–659. 10.1080/10810730.2012.757396 23557148

[B12] Cohen J. Cohen P. West S. G. Aiken L. S. (2003). *Applied Multiple Regression/Correlation Analysis For The Behavioral Sciences*, 3rd Edn. Hillsdale, NJ: Erlbaum.

[B13] Cropanzano R. Mitchell M. S. (2005). Social exchange theory: an interdisciplinary review. *J. Manag.* 31 874–900. 10.1177/0149206305279602

[B14] Dodel M. Mesch G. (2018). Inequality in digital skills and the adoption of online safety behaviors. *Inf. Commun. Soc.* 21 712–728. 10.1080/1369118x.2018.1428652

[B15] Fischer C. Malycha C. P. Schafmann E. (2019). The influence of intrinsic motivation and synergistic extrinsic motivators on creativity and innovation. *Front. Psychol.* 10:137. 10.3389/fpsyg.2019.00137 30778313 PMC6369195

[B16] Giesen C. G. Schmidt J. R. Rothermund K. (2020). The law of recency: an episodic stimulus-response retrieval account of habit acquisition. *Front. Psychol.* 10:2927. 10.3389/fpsyg.2019.02927 32010017 PMC6974578

[B17] Guo S. Guo X. Fang Y. Vogel D. (2017). How doctors gain social and economic returns in online health-care communities: a professional capital perspective. *J. Manag. Inf. Syst.* 34 487–519. 10.1080/07421222.2017.1334480

[B18] Ha J. F. Longnecker N. (2010). Doctor-patient communication: a review. *Ochsner J.* 10 38–43. 21603354 PMC3096184

[B19] Hardey M. (2001). ‘E-health’: the internet and the transformation of patients into consumers and producers of health knowledge. *Inf. Commun. Soc.* 4 388–405. 10.1080/713768551

[B20] Hayes A. F. Matthes J. (2009). Computational procedures for probing interactions in OLS and logistic regression: SPSS and SAS implementations. *Behav. Res. Methods* 41 924–936. 10.3758/brm.41.3.924 19587209

[B21] Honkanen P. Olsen S. O. Verplanken B. (2005). Intention to consume seafood-the importance of habit. *Appetite* 45 161–168. 10.1016/j.appet.2005.04.005 16011859

[B22] Houston M. J. Rothschild M. L. (1977). *A Paradigm For Research On Consumer Involvement*: Graduate School of Business. Madison, WI: University of Wisconsin-Madison.

[B23] Johnston A. C. Worrell J. L. Di Gangi P. M. Wasko M. (2013). Online health communities: an assessment of the influence of participation on patient empowerment outcomes. *Inf. Technol. People* 26 213–235. 10.1108/itp-02-2013-0040

[B24] Kafouros M. Wang C. Piperopoulos P. Zhang M. (2015). Academic collaborations and firm innovation performance in China: the role of region-specific institutions. *Res. Policy* 44 803–817. 10.1016/j.respol.2014.11.002

[B25] Kessler I. Heron P. Dopson S. (2015). Professionalization and expertise in care work: the hoarding and discarding of tasks in nursing. *Hum. Resour. Manag.* 54 737–752. 10.1002/hrm.21695

[B26] Kim A. J. Johnson K. K. (2016). Power of consumers using social media: examining the influences of brand-related user-generated content on Facebook. *Comput. Hum. Behav.* 58 98–108. 10.1016/j.chb.2015.12.047

[B27] Kim H.-S. Mrotek A. (2016). A functional and structural diagnosis of online health communities sustainability: a focus on resource richness and site design features. *Comput. Hum. Behav.* 63 362–372. 10.1016/j.chb.2016.05.004

[B28] Kuang L. Huang N. Hong Y. Yan Z. (2019). Spillover effects of financial incentives on non-incentivized user engagement: evidence from an online knowledge exchange platform. *J. Manage. Inform. Syst.* 36 289–320. 10.1080/07421222.2018.1550564

[B29] Kvedar J. Coye M. J. Everett W. (2014). Connected health: a review of technologies and strategies to improve patient care with telemedicine and telehealth. *Health Aff.* 33 194–199. 10.1377/hlthaff.2013.0992 24493760

[B30] Lee C. Y. Wu H. L. Pao H. W. (2014). How does R&D intensity influence firm explorativeness? Evidence of R&D active firms in four advanced countries. *Technovation* 34 582–593. 10.1016/j.technovation.2014.05.003

[B31] Li Y. Chang Y. (2012). “An integrated model of virtual communities continuous participation,” in *Proceedings of the International Conference on Information Management, Innovation Management and Industrial Engineering* (Piscataway, NJ: IEEE). 10.1109/iciii.2012.6339779

[B32] Li Y. Ma X. Song J. Yang Y. Ju X. (2019). Exploring the effects of online rating and the activeness of physicians on the number of patients in an online health community. *Telemed. E Health* 25 1090–1098. 10.1089/tmj.2018.0192 30676279

[B33] Li Y.-M. Liao T.-F. Lai C.-Y. (2012). A social recommender mechanism for improving knowledge sharing in online forums. *Inf. Process. Manag.* 48 978–994. 10.1016/j.ipm.2011.10.004

[B34] Liu S. Zhang M. Gao B. Jiang G. (2020). Physician voice characteristics and patient satisfaction in online health consultation. *Inf. Manag.* 57:103233. 10.1016/j.im.2019.103233

[B35] Liu X. Guo X. Wu H. Wu T. (2016). The impact of individual and organizational reputation on physicians’ appointments online. *Int. J. Electron. Commer.* 20 551–577. 10.1080/10864415.2016.1171977

[B36] Liu Y. Ren C. Shi D. Li K. Zhang X. (2020). Evaluating the social value of online health information for third-party patients: is uncertainty always bad? *Inf. Process. Manag.* 57:102259. 10.1016/j.ipm.2020.102259

[B37] Liu Y. Zhang X. Zhang Y. Qiu C. (2019). “Research on influencing factors of consumer shopping behavior in online shopping festival,” in *Proceeddings of the Annual Conference of the Society for Management and Economics* (London: AEE Science), 35–42. 10.35532/JSSS.V4.007

[B38] Liu Z. Jansen B. J. (2017). Identifying and predicting the desire to help in social question and answering. *Inf. Process. Manag.* 53 490–504. 10.1016/j.ipm.2016.05.001

[B39] Luo P. Chen K. Wu C. Li Y. (2018). Exploring the social influence of multichannel access in an online health community. *J. Assoc. Inf. Sci. Tech.* 69 98–109. 10.1002/asi.23928

[B40] Luo P. Wang C. Guo F. Luo L. (2021). Factors affecting individual online rumor sharing behavior in the COVID-19 pandemic. *Comput. Hum. Behav.* 125:106968. 10.1016/j.chb.2021.106968 34334932 PMC8314969

[B41] Meng F. Zhang X. Liu L. Ren C. (2021). Converting readers to patients? From free to paid knowledge-sharing in online health communities. *Inf. Process. Manag.* 58:102490. 10.1016/j.ipm.2021.102490

[B42] Meyer K. E. Van Witteloostuijn A. Beugelsdijk S. (2017). What’s in a p? Reassessing best practices for conducting and reporting hypothesis-testing research. *J. Int. Bus. Stud.* 48 535–551. 10.1057/s41267-017-0078-8

[B43] Oo Tha K. K. (2014). “What drives continued sharing knowledge in the electronic network of practice: the case of Wikipedia,” in *Proceedings of the Twentieth Americas Conference on Information Systems*, Savannah.

[B44] Ouellette J. A. Wood W. (1998). Habit and intention in everyday life: the multiple processes by which past behavior predicts future behavior. *Psychol. Bull.* 124 54–74. 10.1037/0033-2909.124.1.54

[B45] Pan S. L. Zhang S. (2020). From fighting COVID-19 pandemic to tackling sustainable development goals: an opportunity for responsible information systems research. *Int. J. Inf. Manag.* 55:102196. 10.1016/j.ijinfomgt.2020.102196 32836647 PMC7338030

[B46] Papadopoulos T. Stamati T. Nopparuch P. (2013). Exploring the determinants of knowledge sharing via employee weblogs. *Int. J. Inf. Manag.* 33 133–146. 10.1016/j.ijinfomgt.2012.08.002

[B47] Parasuraman A. Zeithaml V. A. Berry L. L. (1985). A conceptual model of service quality and its implications for future research. *J. Mark.* 49 41–50. 10.1177/002224298504900403

[B48] Park J. H. Gu B. Leung A. C. M. Konana P. (2014). An investigation of information sharing and seeking behaviors in online investment communities. *Comput. Hum. Behav.* 31 1–12. 10.1016/j.chb.2013.10.002

[B49] Park J. Gabbard J. L. (2018). Factors that affect scientists’ knowledge sharing behavior in health and life sciences research communities: differences between explicit and implicit knowledge. *Comput. Hum. Behav.* 78 326–335. 10.1016/j.chb.2017.09.017

[B50] Reichl P. Kurtansky P. Fabini J. Stiller B. (2006). “A stimulus-response mechanism for charging enhanced quality-of-user experience in next generation all-IP networks,” in *Proceedings of The XIII Conferencia Latino-Ibero-Americana de Investigación de Operaciones* (Uruguay: Montevideo). 10.5167/uzh-67539

[B51] Samila S. Sorenson O. (2010). Venture capital as a catalyst to commercialization. *Res. Policy.* 39 1348–1360. 10.1016/j.respol.2010.08.006

[B52] Straub D. Limayem M. Karahanna-Evaristo E. (1995). Measuring system usage: implications for IS theory testing. *Manag. Sci.* 41 1328–1342. 10.1287/mnsc.41.8.1328 19642375

[B53] Van Deursen A. J. Van Dijk J. A. Peters O. (2011). Rethinking Internet skills: the contribution of gender, age, education. Internet experience, and hours online to medium-and content-related Internet skills. *Poetics* 39 125–144. 10.1016/j.poetic.2011.02.001

[B54] Van Oerle S. Mahr D. Lievens A. (2016). Coordinating online health communities for cognitive and affective value creation. *J. Serv. Manag.* 27 481–506. 10.1108/josm-09-2015-0264

[B55] Wan X. Sanders N. R. (2017). The negative impact of product variety: forecast bias, inventory levels, and the role of vertical integration. *Int. J. Prod. Econ.* 186 123–131. 10.1016/j.ijpe.2017.02.002

[B56] Watson J. B. (1913). Psychology as the behaviorist views it. *Psychol. Rev.* 20 158–177. 10.1037/h0074428

[B57] Wiener S. Lee C.-Y. (2020). Multi-talker speech promotes greater knowledge-based spoken Mandarin word recognition in first and second language listeners. *Front. Psychol.* 11:214. 10.3389/fpsyg.2020.00214 32161560 PMC7052525

[B58] Wu H. Lu N. (2017). Online written consultation, telephone consultation and offline appointment: an examination of the channel effect in online health communities. *Int. J. Med. Inform.* 107 107–119. 10.1016/j.ijmedinf.2017.08.009 29029686

[B59] Wu W. Wang H. Lee H.-Y. Lin Y.-T. Guo F. (2019). How machiavellianism, psychopathy, and narcissism affect sustainable entrepreneurial orientation: the moderating effect of psychological resilience. *Front. Psychol.* 10:779. 10.3389/fpsyg.2019.00779 31110485 PMC6499190

[B60] Wu Y. Po H. (2016). *China’s Changing Pharmaceutical E-Commerce Market.* Beijing: Deloitte China.

[B61] Xu X. Li Q. Peng L. Hsia T.-L. Huang C.-J. Wu J.-H. (2017). The impact of informational incentives and social influence on consumer behavior during Alibaba’s online shopping carnival. *Comput. Hum. Behav.* 76 245–254. 10.1016/j.chb.2017.07.018

[B62] Yan L. Tan Y. (2014). Feeling blue? Go online: an empirical study of social support among patients. *Inf. Syst. Res.* 25 690–709. 10.1287/isre.2014.0538 19642375

[B63] Yan Z. Wang T. Chen Y. Zhang H. (2016). Knowledge sharing in online health communities: a social exchange theory perspective. *Inf. Manag.* 53 643–653. 10.1016/j.im.2016.02.001

[B64] Yang H. Ju X. (2016). “Investigating the influences of motivators on physician contribution behaviors in online health community: Offline status as a moderator,” in *Proceedings of the PACIS.* https://aisel.aisnet.org/pacis2016/11 (accessed August 23, 2016).

[B65] Yang H. Guo X. Wu T. Ju X. (2015b). Exploring the effects of patient-generated and system-generated information on patients’ online search, evaluation and decision. *Electron. Commer. Res. Appl.* 14 192–203. 10.1016/j.elerap.2015.04.001

[B66] Yang H. Guo X. Wu T. (2015a). Exploring the influence of the online physician service delivery process on patient satisfaction. *Decis. Support Syst.* 78 113–121. 10.1016/j.dss.2015.05.006

[B67] Zhang X. Guo X. Lai K.-H. Yi W. (2019b). How does online interactional unfairness matter for patient–doctor relationship quality in online health consultation? The contingencies of professional seniority and disease severity. *Eur. J. Inform. Syst.* 28 336–354. 10.1080/0960085x.2018.1547354

[B68] Zhang X. Fang Y. He W. Zhang Y. Liu X. (2019a). Epistemic motivation, task reflexivity, and knowledge contribution behavior on team wikis: a cross-level moderation model. *J. Assoc. Inf. Sci. Tech.* 70 448–461. 10.1002/asi.24129

[B69] Zhang X. Liu S. Deng Z. Chen X. (2017b). Knowledge sharing motivations in online health communities: a comparative study of health professionals and normal users. *Comput. Hum. Behav.* 75 797–810. 10.1016/j.chb.2017.06.028

[B70] Zhang X. Guo X. Lai K.-H. Yin C. Meng F. (2017a). From offline healthcare to online health services: the role of offline healthcare satisfaction and habits. *J. Electron. Commer. Res* 18 138–154.

[B71] Zhang X. Liu L. Meng F. (2021). User psychology and behavior regarding healthcare IT. *Front. Psychol.* https://www.frontiersin.org/research-topics/23917/user-psychology-and-behavior-regarding-healthcare-it 10.3389/fpsyg.2022.1068517 PMC9706179 36457919

[B72] Zhao H. Fu S. Chen X. (2020). Promoting users’ intention to share online health articles on social media: the role of confirmation bias. *Inf. Process. Manag.* 57:102354. 10.1016/j.ipm.2020.102354 32834400 PMC7368841

